# New Developments in Psychiatric Classification: A Transdiagnostic Approach

**DOI:** 10.7759/cureus.84580

**Published:** 2025-05-21

**Authors:** Camila Farías Venegas, Luis Felipe Varela Espinoza, Christopher Ramírez Matta, Bernardo Barra Cañas, Mónica Araneda Maldonado, Alejandro Sánchez Oñate

**Affiliations:** 1 Psychiatry, Universidad Andrés Bello, Santiago, CHL; 2 Psychiatry, Universidad de Los Andes, Chile, Santiago, CHL; 3 Psychology, Instituto de Bienestar Socioemocional, Universidad del Desarrollo, Concepción, CHL

**Keywords:** classification, diagnosis, nosology, psychiatry, transdiagnostic

## Abstract

Psychiatric nosology, traditionally represented by the International Classification of Diseases (ICD) and the Diagnostic and Statistical Manual of Mental Disorders (DSM), is based on categorical diagnoses that have not met the current needs of clinicians. The so-called transdiagnostic approach appears to offer promise for classifying mental disorders. In the present study, our objective was to conduct a descriptive analysis of this approach in psychiatry based on the current literature.

This study is a narrative review that utilized a bibliographic search for original and review articles on the transdiagnostic approach in psychiatry. The following keywords were used: "transdiagnostic", "psychiatry", "diagnosis", "approach", and "classification." Searches were conducted in PubMed, ScienceDirect, Scielo, the Cochrane Library, PsycINFO, Lilacs, and Hinari. Articles in English and Spanish with publication dates between 2003 and 2024 were included. A total of 261 records were identified for screening, and 104 were retrieved for full review.

The DSM and ICD are systems that define aspects of our conceptualization of mental health. More recently, however, transdiagnostic perspectives have gained prominence, particularly through frameworks such as the RDoC (Research Domain Criteria) and HiTOP (Hierarchical Taxonomy of Psychopathology). The RDoC stands out for guiding research on the neurobiological bases of psychopathology. HiTOP is based on data and a hierarchy that conceptualizes psychopathology as a set of transdiagnostic dimensions and spectra. Both approaches, with their strengths and weaknesses, could be an interface and the basis for a new nosology in psychiatry.

There is consensus on the need for a new operational framework for classification. The transdiagnostic approach could be the contemporary answer to this premise. The challenge is to develop further research and standardize the definition of the construct.

## Introduction and background

Contemporary psychiatric nosology has traditionally been represented by two main classification systems: the International Classification of Diseases (ICD), issued by the World Health Organization, and the Diagnostic and Statistical Manual of Mental Disorders (DSM), developed by the American Psychiatric Association [1,2]. Both are based on categorical diagnoses that have allowed the unification and standardization of obtaining the symptoms of mental disorders [1,2].

In its quest to improve classification in the study of psychiatry, the DSM-5 incorporated some changes in its structure, some of them being; the elimination of the multiaxial system, greater preponderance in the role of the life cycle within the organization of its chapters compared to previous manuals, and even, to a certain extent, the inclusion of a more dimensional view when describing the severity of the pathologies (mild, moderate and severe) in different clinical pictures (such as major depressive disorder) [3].

Despite the contributions sought by these manuals, the categorical approach has shown over time that it polarizes symptoms into all-or-nothing phenomena, induces the examiner to conduct an interview by reducing the phenomenological product, and selects information that does not always have adequate prioritization in the diagnostic process [4].

In clinical practice, it is observed that the expression of mental disorders is heterogeneous, being diagnosed based on transversal and longitudinal symptom profiles. In addition to the above, it is observed that different symptom patterns can lead to the same diagnosis and overlap (within and between different classes of diagnoses), added to the presence of comorbidities, leading to diverse clinical manifestations and trajectories [5].

Reinforcing the above, in recent years, there has been a significant increase in the number of empirical and meta-analytic studies that seek and show the neural, physiological, and/or psychological deficiencies that are present in supposedly different diagnostic categories, rather than those that might be specific to one category [6,7].

In response to all of the above, the so-called transdiagnostic approach proposes a new look at the model regarding how we classify in psychiatry. This dimensional approach suggests a kind of common foundation on which diagnoses are built, taking into account two aspects that have been relevant up to now: the high comorbidity and the heterogeneity of mental disorders [8].

Although the term “transdiagnostic” has been used more often in recent years, there are conceptual differences in its use. In 2019, the largest systematic review to date on the transdiagnostic approach was conducted, finding that most of these studies referring to transdiagnostic have focused on evaluating depressive and anxiety disorders, and that most of the studies found, in reality, what they defined as transdiagnostic did not meet the criteria for a truly transdiagnostic approach [2].

Therefore, this work proposes to descriptively analyze the transdiagnostic approach in psychiatry according to the current literature, as well as to describe the main existing approaches on the unification of transdiagnostic symptomatology in psychiatry.

## Review

Methods

This study is a narrative review that utilized a bibliographic search for original and review articles on the transdiagnostic approach in psychiatry. The following keywords were used: "transdiagnostic", "psychiatry", "diagnosis", "approach", and "classification". Searches were conducted in PubMed, Science Direct, Scielo, the Cochrane Library, PsycINFO, Lilacs, and Hinari. Articles in English and Spanish with publication dates between 2003 and 2024 were included. Duplicate articles, articles in another language, articles not freely available in full text, and articles unrelated to the research topic were excluded.

**Figure 1:**
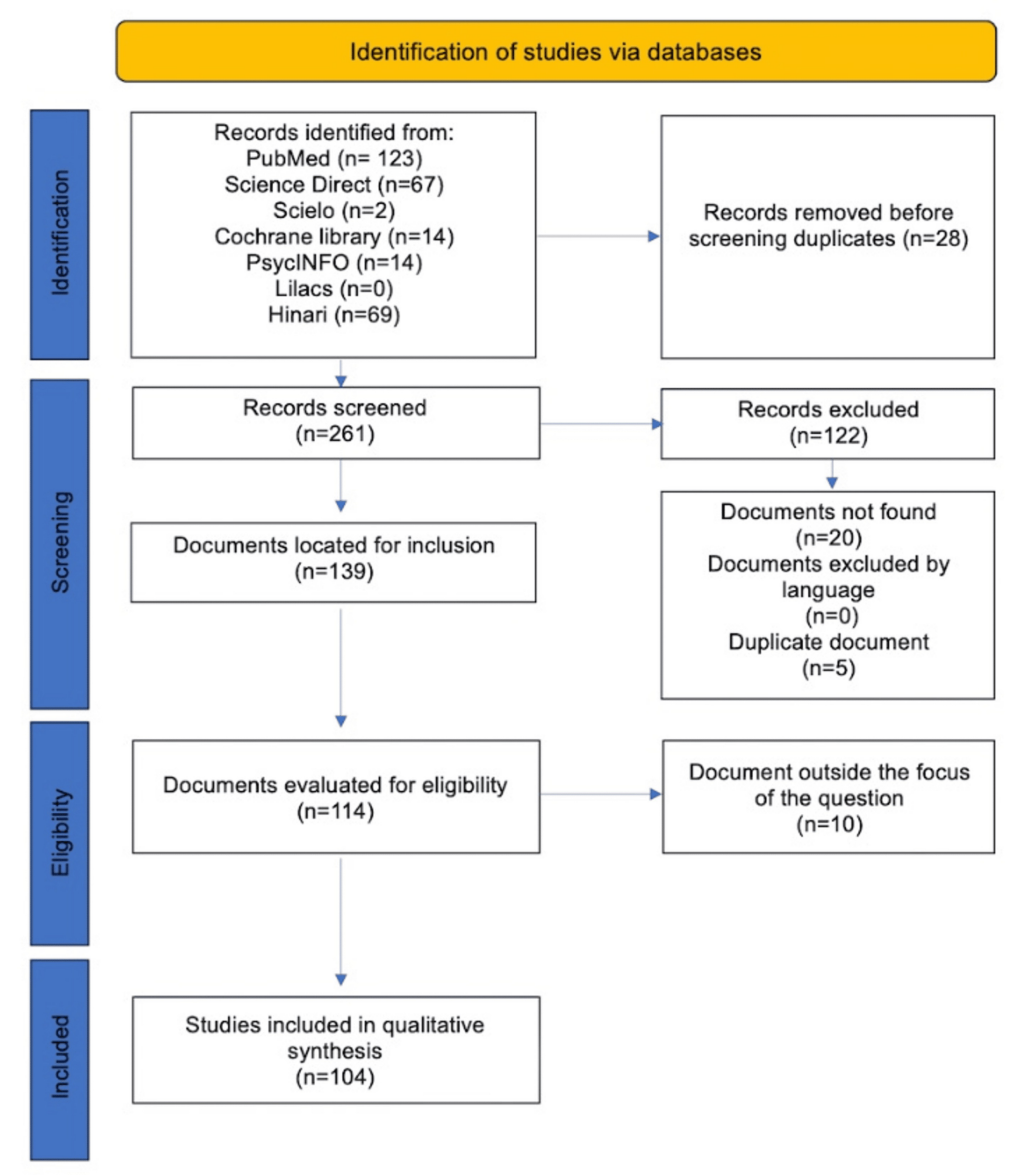
The selection process of articles used in this study. Adopted from the Items for Systematic Reviews and Meta-Analysis (PRISMA)

Two reviewers separately screened the titles and abstracts, and the full texts of the selected publications were then analyzed. During this review, in the event of disagreement, consensus was reached through discussion with a third author. A total of 261 records were identified for screening, and 104 were retrieved for full-text review. These records were synthesized and presented narratively in this study. The method used to select the articles is shown in Figure 1.

Exploring psychiatric nosology approaches

When we refer to “nosology” (a set of classification categories), we do so in broader terms than just categories [9]. We use the concept with an eye to theory that is intrinsically linked to the nature of what we are classifying. In this context, an important question arises in the words of Jablensky: “Are we dealing with discrete, discontinuous entities, or with continuous, graded phenomena to which we can apply a sliding rule of thresholds to separate “pathology” from “normal variation” and determine the need for treatment?” [9].

Classically, there are two classification approaches: the categorical one (predominant in history) and the dimensional one (complement of contemporary spectral models such as the affective spectrum of Akiskal or the schizophrenic spectrum of Parnas) [10]. The categorical model assumes that a disorder is either present or not, based on how closely a case matches an archetypal clinical presentation. Yet, when a patient exhibits symptoms corresponding to more than one condition, for instance, panic disorder and social anxiety, questions arise: are these two distinct syndromes, or does their co-occurrence reflect a unique diagnostic entity? Evidence suggests that psychiatric conditions often coexist, casting doubt on strict categorical distinctions [11-14]. 

On the other hand, dimensional approaches propose that symptoms of a disorder lie along a spectrum, ranging from normal, typical functioning to severe impairment. In this view, disorders can be characterized by the number of symptoms present (e.g., at least three of six criteria for generalized anxiety disorder) and the intensity of specific symptom clusters. A diagnostic threshold is when a condition qualifies as a disorder, converting this continuum of symptoms into a categorical classification. This threshold is often established based on expert consensus or clinical judgment [10].

Descriptive analysis of the transdiagnostic approach in psychiatry according to the literature

The DSM and ICD provide an organizing framework for virtually all core psychiatry texts, offering a standardized framework that guides the recognition, diagnosis, and treatment of mental disorders across diverse healthcare systems worldwide [15,16]. Current research aims to improve psychiatric nosology, but even with all the advances of the recent century, we still have two major problems: high rates of misdiagnosis and available categorical classification systems with low clinical utility for prognosis and treatment [17,18]. 

The first point regarding diagnostic accuracy presents two epistemic problems: on the one hand, the question arises as to whether the classification system used reflects the desired organizational principle, and whether the classification method is accurate when applying it. Expressed another way, the method could fail, even if the “classification system” were well designed [19]. In this case, precision should be oriented towards individual cases; for example, even within the well-structured DSM system, if should be questioned if a a clinician has correctly diagnosed a patient with a depressive episode. A good classification system should satisfy both postulates [19].

Although numerous studies have explored the genetic basis of mental disorders, we still lack a comprehensive explanation of their origins [20]. Furthermore, there is an approximate 40% comorbidity rate in mental disorders, suggesting that molecular mechanisms are shared with common environmental stressors [21,22]. Both diagnostic classification and etiology are strongly interdependent [19,23]. An alternative perspective has emerged from empirical research, emphasizing the patterns in signs, symptoms, and behaviors that can be systematically assessed, leading to classification models grounded in data [24].

The transdiagnostic approach is largely based on an epistemological error [2]. Both the DSM and the ICD have simplified psychopathological phenomena, reducing complex symptoms to phenomenological primitives. For example, there is only one type of depressive state, one type of delirium, and all these states are assumed to share the same phenomenological structure when observed in different mental disorders [2,3].

In this scenario, transdiagnostic therapy appeared in 2003, when Fairburn et al. described a mental health treatment in the context of cognitive behavioral therapy (CBT) for eating disorders [25]. In 2004, Harvey et al. used studies of people with classified disorders to show that the psychological processes that maintain distress are shared across diagnoses [26]. In the same year, the first evaluation of a "transdiagnostic" group intervention for anxious symptoms in depression was published by Norton et al. [27].

Regarding the use of the term, Harvey et al. in 2011 highlighted the differences between constructs that are “mechanistically transdiagnostic” and those that are “descriptively transdiagnostic” [28]. The concept of “mechanistically transdiagnostic” indicates that it plays a causal role across various forms of psychopathology. In comparison, “descriptively transdiagnostic” refers to constructs that appear across different disorders, independent of the underlying mechanisms. A typical example is low self-esteem, which is commonly observed in multiple clinical conditions such as depression and schizophrenia. While low self-esteem may fit the descriptively transdiagnostic profile, there is currently no comprehensive theoretical model that explains its contribution to the onset or persistence of these disorders [29].

Mechanistically transdiagnostic constructs reflect shared risk factors that increase the likelihood of an individual developing multiple health conditions, due to common underlying mechanisms that contribute to symptom expresión across different disorders [29]. Understanding these constructs may enhance the development of more streamlined and effective therapies.

New transdiagnostic approaches in psychiatry: RDoC and HiTOP

Aligned with the emergence of mechanistically transdiagnostic constructs, the US National Institute of Mental Health (NIMH) reoriented its research agenda, distancing itself from traditional diagnostic classifications in order to identify therapeutic targets and enhance treatment effectiveness. With this objective in mind, the Research Domain Criteria (RDoC) initiative was established in 2009. Its aim is to organize the scientific evidence on mental disorders in order to enhance treatment efficacy by promoting research into the neurobiological underpinnings of psychopathology structured around biobehavioral dimensions and contributing to the refinement of psychiatric classification systems [30,31].

The RDoC framework was designed to address key limitations of categorical diagnostic models, including the dimensional character of psychopathology, the insufficient focus on its underlying mechanisms, and the marked heterogeneity observed within diagnostic groups. It promotes investigation into core biobehavioral systems, such as cognitive and social functioning, that cut across conventional diagnostic divisions, and emphasizes their relationship with behavioral expressions over rigid diagnostic labels [31]. 

RDoC does not function as a diagnostic system, nor does it position itself as such. Rather than offering labels or formal classifications, it serves as a model for organizing translational research in psychiatry. It is based on systems theory applied to neuroscience, establishing a methodology for linking techniques and sources of information to research targets. The proposal is that research in psychopathology should be oriented toward the study of systems through different sources of evidence. The main differences between RDoC and current categorical systems are that RDoC follows a data-driven strategy and from there to the construct; considers the importance of endophenotypes (internal phenotypes observable through biochemical measurements, imaging or laboratory tests) and starts from an integrative vision, intended to provide a structure that gives equal weight to the different levels of analysis [31-32]. 

In 2015, a group of psychologists and psychiatrists with a common interest in developing quantitative models of psychiatric classification came together to establish a consortium aimed at advancing the Taxonomy of Psychopathology (HiTOP). This framework offers a statistically grounded, multilevel alternative that views mental disorders as dimensions structured within progressively broader transdiagnostic spectra. HiTOP´s multilevel structure captures psychopathology across varying degrees of generality and specificity. At the narrowest level are components, defined as symptom clusters or maladaptive behaviors (e.g., sleep disturbances); components that tend to co-occur form broader syndromes (e.g., generalized anxiety); similar síndromes are grouped into subfactors (e.g., fear); and these, in turn, are organized into higher-order spectra (e.g., externalizing) [31].

Broad, overarching dimensions have also been proposed, including the general factor of psychopathology, commonly referred to as the p-factor. This concept, introduced by psychologists Caspi and Moffitt in 2014, suggests that mental disorders may share a common, rather than distinct or disorder-specific, etiological foundation [33]. Within the HiTOP framework, six major spectra have been defined to date: internalizing, disinhibited externalizing, antagonistic externalizing, thought disorder, detachment, and somatoform. These are further subdivided into seven empirically derived subfactors [31].

Currently, the model incorporates the most common forms of psychopathology, but there are still others not included, such as most neurodevelopmental problems. Its dimensional structure has demonstrated empirical support in terms of etiological relevance, prognostic value, and clinical usefulness. However, specific biological bases have not been formally incorporated into the model [34].

Some considerations emerge when analyzing HiTOP, including the possibility that the biological and behavioral features related to specific dimensions may vary across time. As a result, studies conducted at different developmental stages could yield divergent associations. Although HiTOP holds promise for enhancing treatment relevance, there is currently no conclusive evidence that applying this model in clinical contexts leads to improved outcomes or effectively integrates with treatment development efforts [31].

HiTOP frames clinical presentations through psychopathological profiles rather than relying on traditional categorical diagnoses. Interpreting these broader profiles may offer a more streamlined and conceptually coherent alternative to listing multiple comorbid conditions. However, as with the RDoC framework, one challenge lies in the fact that administrative funding mechanisms in much of the world are tied to ICD-based diagnosis codes [34,35]. To address this limitation, the HiTOP Clinical Translation Workgroup created an alternative tool--the HiTOP-ICD (freely accessible upon request)--which aims to support clinical application by aligning HiTOP domains with ICD codes for administrative use [35].

Key similarities and differences between RDoC and HiToP

When comparing both approaches, different analyses emerge. Among their main similarities are that both move away from diagnostic categories and are created in a dynamic and evolutionary work with a view to being reviewed by groups of experts constantly over time [31-36]. On the other hand, regarding their differences, the debate is greater (Table 1).

**Table 1:** Main differences between HiToP and RDoC. Table prepared by the authors based on a synthesis of key plubications on HiTOP and RDoC [31,34]. HiTOP: Hierarchical Taxonomy of Psychopathology; RDoC: Research Domain Criteria

Category	HiToP	RDoC
Classification type	Hierarchical dimensions.	Non-hierarchical dimensions.
Objective	To reorganize and reconceptualize clinical classification to improve prevention and treatment.	Neurobiological study purposes based on psychopathological dimensions.
Basis of its construction	Based on factor analysis and latent class analysis, a quantitative approach.	Based on expert study and observation, a qualitative approach.
Addressing the limitations of categorical systems	Addresses the dimensional nature of psychopathology, widespread comorbidity, heterogeneity within the disorder, and symptom overlap.	Addresses the dimensional nature of psychopathology, the lack of consideration of underlying foundations, and the heterogeneity within the disorder.
Content	It focuses on symptoms, signs, diagnosis, and behavior.	It covers everything from genes to the brain and behavior, with an emphasis on neurobiology.
Limitations	It includes clinical elements with a description of psychopathological phenomena, but does not take the neurobiological foundations into account.	It is a research framework with limited application to the clinic.

In this context, the possible link between RDoC and HiToP arises [31]. In this interaction, researchers on the subject have observed the potential to lay the foundations of a new nosology in psychiatry.

## Conclusions

It has been argued that the categorical approach adopted by the DSM and ICD stands at odds with current clinical and research findings, potentially limiting our understanding of mental disorders, as well as their assessment and treatment. These systems render the clinical phenomenological product invisible and fail to reflect the modulation and multiple interactions produced by an individual's life experiences, including genetics.

There are questions that have been and continue to be debated: should psychiatric disorders be grouped by symptoms or by causes? Should disorders be defined by subjective experience or by objective criteria? Are psychiatric disorders distinct entities or points along a spectrum? While the promise of the modern approach may seem to be the answer, the transdiagnostic approach in psychiatry, in turn, is conceptually incoherent, diverse, and focused on a limited subset of mental disorders. 
